# Nursing practice of routine gastric aspiration in preterm infants and its link to necrotizing enterocolitis: is the practice still clinically relevant?

**DOI:** 10.1186/s12912-024-01994-x

**Published:** 2024-05-17

**Authors:** Osama Mohamed Elsayed Ramadan, Majed Mowanes Alruwaili, Abeer Nuwayfi Alruwaili, Nadia Bassuoni Elsharkawy, Enas Mahrous Abdelaziz, Mohammed Elsayed Zaky, Marwa Mamdouh shaban, Mostafa Shaban

**Affiliations:** 1 College of Nursing, Jouf University, Sakaka, Saudi Arabia 72388; 2 Maternal and Newborn Health Nursing Department, Faculty of Nursing, Cairo University, Cairo, Egypt; 3 Psychiatric Mental Health Nursing Department, Faculty of Nursing, Cairo University, Cairo, Egypt; 4 Medical Surgical Nursing Department, Faculty of Nursing, Cairo University, Cairo, Egypt; 5 Lecturer of Community Health Nursing, Faculty of Nursing, Cairo University, Cairo, Egypt; 6 Geriatric Nursing Department, Faculty of Nursing, Cairo University, Cairo, Egypt

**Keywords:** Necrotizing enterocolitis, Preterm infants, Gastric aspiration, Gastric residuals, Feed tolerance, Evidence-based practice

## Abstract

The practice of routine gastric residual aspiration in preterm infants remains controversial, with conflicting evidence regarding its impact on necrotizing enterocolitis (NEC). As front-line caregivers, nurses play a vital role in gastric aspiration procedures and must be informed by evidence. This quasi-experimental nursing study aimed to assess whether gastric aspiration is clinically relevant in reducing the risk of NEC in preterm infants.

A total of 250 preterm infants from two NICUs in Egypt were allocated to the gastric aspiration (*n* = 125) and non-aspiration (*n* = 125) groups. Feeding practices, gastric residuals, and incidence/severity of NEC were compared between groups according to modified Bell’s criteria. Risk factors were analyzed using multivariate regression. There were no significant baseline differences between the groups. The gastric residual attributes and feeding outcomes did not differ substantially from aspiration. The overall incidence of NEC was 14–15%, with no significant differences in the odds of onset or progression of NEC by stage between the groups. Lower gestational age and birth weight emerged as stronger predictors of NEC. Routine gastric aspiration does not appear to directly prevent or reduce the severity of NEC in this population. Although gastric residuals retain clinical importance, study findings question assumptions that aspiration protects against NEC and informs nursing practice. Evidence-based feeding protocols must continually evolve through ongoing research on modifiable risk factors for this devastating intestinal disease in preterm infants.

## Introduction

Preterm birth, defined as delivery before 37 weeks of gestation, poses a significant challenge in neonatal nursing due to underdeveloped organ systems and increased susceptibility to complications [1–4]. The care of preterm infants requires a collaborative, interdisciplinary approach involving neonatal nurses, physicians, and other healthcare professionals to optimize outcomes and mitigate the risk of complications. The immature organ systems of preterm infants leave them vulnerable to a myriad of complications, necessitating specialized care in neonatal intensive care units (NICUs) [5–8].

Among the many challenges faced by preterm infants, achieving optimal nutrition is recognized as a critical factor in ensuring their growth, development, and general health [9–14]. However, providing adequate nutrition is complicated by the prevalence of feeding difficulties in this population [15, 16], such as poorly coordinated sucking and swallowing reflexes [17], as well as the looming risk of serious complications, particularly necrotizing enterocolitis (NEC) [5, 18–22]. In addition to poorly coordinated sucking and swallowing reflexes, preterm infants often experience feeding intolerance, which can manifest as increased gastric residuals and may prompt the use of gastric residual aspiration to assess feeding readiness and prevent complications [23].

NEC is a life-threatening gastrointestinal emergency that disproportionately affects preterm infants, with potentially devastating consequences [15, 24]. The condition is characterized by inflammation and necrosis of the intestinal tissue, leading to high rates of morbidity and mortality [25–27]. Despite advances in neonatal care, the precise etiology of NEC remains elusive [28–31], although several risk factors have been identified, including prematurity, formula feeding, and aberrant gut microbial colonization [23, 32]. The complex multifactorial nature of NEC underscores the importance of early detection and prompt intervention to mitigate its impact on preterm infants [33, 34]. In this context, neonatal nurses play a crucial role in preventing and managing NEC through meticulous monitoring, clinical evaluation, and implementing evidence-based feeding protocols [35–37].

Nurses in NICUs are familiar with the prevalence of NEC and its significant effects on the care and results of premature infants [38–40]. Routine practices such as measuring the residual volume of the stomach (GRV) for the diagnosis and prevention of complications associated with NEC highlight the crucial and practical role of nursing in neonatal care [28–31]. One of the most common practices in NICUs around the world is the routine aspiration of gastric residuals prior to feeding as a means of assessing feeding tolerance, securing feeding tube placement, and preventing potential complications [1, 41–45]. This procedure involves aspiration of the contents of the stomach through a feeding tube to assess the volume and characteristics of the residuals, which have been traditionally used to guide feeding decisions, despite ongoing debate about their clinical significance and reliability as indicators of digestive function and feeding readiness [42, 46, 47].

A growing body of research has sought to elucidate the relationship between gastric residual aspiration and the development of NEC, producing contradictory and inconclusive results [48, 49]. Some studies suggest that routine aspiration of gastric residuals can disrupt the delicate balance of the developing gut microbiome, potentially increasing the risk of NEC [15, 50–55]. On the contrary, other investigations have failed to demonstrate a significant association between gastric residual aspiration and the incidence of NEC [54, 56]. This lack of consensus within the scientific community highlights the urgent need for more research to clarify the role of gastric residual aspiration in the pathogenesis and prevention of NEC [57–59].

Beyond clinical practice, this research has major implications for preterm infant nursing education and policy. This study’s contribution to stomach residual aspiration and NEC understanding helps change the curriculum of neonatal nursing programs, ensuring future nurses have the latest and most evidence-based procedures. This research can also influence clinical recommendations and methods in NICUs worldwide to standardize NEC prevention and care. In summary, this study is crucial to understanding the complex link between residual gastric aspiration and NEC in preterm infants. This research fills a gap in the literature to clarify how this frequent practice contributes to a potentially fatal condition. Our objective is to improve evidence-based neonatal care and provide preterm infants with the best treatment to support their growth, development, and well-being through a comprehensive and rigorous approach. Neonatal nurses must endeavor to understand the problems faced by our most fragile preterm infants, and this study is an essential step toward knowledge and excellence in care.

## Materials and methods

### Research question

Is there a difference in the incidence and severity of necrotizing enterocolitis (NEC) between preterm infants who undergo routine gastric residual aspiration and those who do not?

### Hypothesis

Our central hypothesis is that gastric residual aspiration in preterm infants could influence the incidence of NEC. Specifically, we postulate that:

**H1.*** Preterm Infants who undergo routine gastric residual aspiration have a reduced risk of developing NEC compared to those who do not undergo aspiration.*

**H2.*** Routine assessment of gastric residuals may not provide significant clinical benefits in predicting or preventing complications such as NEC.*

### Design

A quasi-experimental design was used to achieve the objective of the study. Quasi-experiments aim to estimate the causal effects of an intervention on the target population without randomly assigning subjects to a group [60].

### Settings

The first NICU is located at Cairo University Children’s Hospital (El Monira). With a capacity of 40 incubators, it offers complimentary advanced neonatal care to infants throughout Egypt. This unit is segmented into an intermediate care area that houses 15 incubators for secondary treatment and an intensive care section with 25 incubators dedicated to tertiary care. Additional amenities include isolation chambers, breastfeeding support, and outpatient clinics.

On the contrary, the second NICU is located on the fourth floor of the maternity wing of El Manial University Hospital. This unit, equipped with 35 incubators, also provides state-of-the-art neonatal care. It features an intermediate care section with 15 incubators, two intensive care zones (each containing 5 incubators) designed for diverse and infected neonates, immediate postnatal care with 10 incubators, a designated breastfeeding area, and a medication preparation facility.

### Sample

A priori power analysis was performed to determine the target sample size needed to detect a significant difference in the incidence of NEC between the gastric aspiration and non-aspiration groups. Based on previous studies, the incidence of NEC in preterm infants is approximately 10% [61, 62]. We hypothesized that the gastric aspiration intervention could reduce this incidence by 3%, which would be clinically significant given the available sample size. With a power of 80%, an alpha of 0.05, and the recruited sample size of 125 infants per group (250 infants in total), the study is sufficiently powered to detect a 3% reduction in the incidence of NEC between the groups. Power analysis was conducted using G*Power software (version 3.1.9.7) [63].

The software calculated a total sample size of 236 infants, rounded to 250 to account for potential attrition. Therefore, this study aimed to recruit a convenience sample of 250 preterm infants admitted to the NICU of El Manial University Hospital and Elmonira Pediatric Hospital, with a target of 125 infants assigned to each study group. This sample size provides adequate statistical power to detect a clinically significant difference of 3% in the incidence of NEC between the gastric residual aspiration and non-aspiration groups.

- The allocation procedure was as follows:

As infants were admitted to the NICU, they were screened for eligibility based on the predefined inclusion and exclusion criteria. Eligible infants underwent a 48-hour observation period before enrollment. After obtaining informed parental consent, the enrolled infants were allocated to either the gastric aspiration or non-aspiration group as they were recruited. The allocation was quasi-random based on the order of admission to the NICU, assigning approximately half to each study group in an alternating fashion throughout the recruitment period. For example, the first eligible enrolled infant was allocated to the aspiration group, the second to the non-aspiration group, the third to the aspiration group, and so on until the target sample size was achieved in both groups. This allocation order was not completely random but intended to distribute interventions evenly across the recruitment timeframe. The final group allocation was 125 infants in the gastric residual aspiration group and 125 infants in the non-aspiration group.

### Eligibility criteria

#### Inclusion criteria

Subjects were considered if they were preterm infants born at less than 37 weeks of gestation receiving tube feeding (orogastric or nasogastric).

While the inclusion criteria encompassed all preterm infants born before 37 weeks, it is important to note that the study population primarily consisted of infants with lower gestational ages, as evidenced by the reported mean gestational age of 28.5 weeks. This is likely due to the fact that infants with lower gestational ages are more likely to require nasogastric feeding and are at a higher risk of developing feeding-related complications, such as necrotizing enterocolitis. It is important to note that the risk of developing NEC is inversely related to gestational age, with infants born at lower gestational ages being at a higher risk compared to those born at later gestational ages [64–66]. This increased risk is likely due to the greater immaturity of the gastrointestinal tract, immune system, and other organ systems in infants born at earlier gestational ages [66].

#### Exclusion criteria

Subjects with intrauterine growth retardation (birth weight below the 10th percentile for gestational age and sex) or preterm infants with respiratory distress (> 80 breaths/min) are excluded. Other factors could include circulatory instability that requires inotropic treatment, highly suspected early-onset sepsis with a change in clinical general condition, worse peripheral perfusion, and circulatory decompensation before the start of the study (within the first 6 h after admission to the neonatal unit); and gastrointestinal tract malformations such as congenital diaphragmatic hernia and other life-limiting congenital severe malformations, since any of these health problems can affect preterm infant feeding.

### Data collection tools

Detailed clinical information was extracted from infant medical and nursing records using a standardized data collection form. The information collected included:

- Neonatal characteristics: gestational age, birth weight, sex, maternal complications.

- Medical history: antenatal steroids, multiple births.

- Vital signs: heart rate, respiratory rate, oxygen saturation.

- Feeding details: route of enteral feeding (oral vs. nasal tube), type of milk (breast milk or formula) and time to reach full feeds.

- Gastric residuals: volume, consistency, frequency of measurement.

- Complications: suspected or confirmed necrotizing enterocolitis and staging according to modified Bell’s criteria.

The validity and reliability of medical records as a data source have been well-established in previous studies [15, 24]. Data was extracted by trained nurses using an extraction manual to ensure standardized collection. Inter-rater reliability testing between data extractors showed excellent agreement.

Modified Bell’s Staging Criteria [67] was used to diagnose and classify NEC based on clinical, laboratory, and radiographic findings. This classification system has been extensively validated with high sensitivity (95%) and specificity (98%) for identifying NEC infants [67]. Stage progression was also documented. All stages were overread by an independent pediatric gastroenterologist to confirm NEC diagnosis and staging. Inter-rater reliability between nurses and the gastroenterologist was substantial (kappa = 0.82) [68].

A detailed feeding tolerance form was used to record gastric residual volumes, characteristics, and frequency. This form demonstrated excellent validity based on comparisons with actual measured residual volumes (*r* = 0.91). Test-retest reliability over multiple feeds was also high (*r* = 0.88), confirming consistency.

Using validated data collection tools and standardized extraction procedures, we aimed to ensure high-quality data capture to reliably test our study hypotheses related to gastric residuals, feeding, and NEC outcomes. The validity testing and inter-rater reliability help minimize information bias and standardize clinical data interpretation between extractors.

### Ethics approval

Ethical approval was obtained from the Cairo University Faculty of Nursing, before the start of the study (IRB20194041701). Subsequently, written informed consent was obtained from the parents of preterm infants within 48 h and 24 h after the start of feeding. After the parents of eligible premature infants have been informed of the nature of the investigation. They were also assured of the confidentiality of their wards and informed that their participation was voluntary and that they had the right to withdraw at any time.

### Procedure

A systematic recruitment procedure was crucial to successfully conducting our research. The following steps represent the approach we adopted:

#### Participant recruitment

The study population included preterm infants admitted to the El Manial University Hospital NICU and Elmonira Pediatric Hospital in Egypt between January 2022 and April 2023. Infants were assessed for eligibility within 48 h of NICU admission. The inclusion criteria were gestational age < 37 weeks and enteral feeding (oral or nasal tube). Exclusion criteria included intrauterine growth restriction, respiratory distress > 80 breaths/min, circulatory instability requiring inotropes, suspected early-onset sepsis, and significant congenital anomalies. Eligible infants underwent a 48-hour observation period before enrollment. Parents/caregivers of eligible infants were provided with study information and gave written informed consent for participation.

#### Group allocation

Infants enrolled were assigned 1:1 to the gastric residual aspiration or non-aspiration groups when admitted to the NICU. Allocation order was quasi-random based on admission date, with the goal of approximately equal group sizes. The final group sizes were 125 infants in the aspiration group and 125 infants in the non-aspiration group.

#### Feeding interventions

In the aspiration group, gastric residuals were aspirated through an orogastric or nasogastric tube and measured in milliliters before each feeding. The appearance of the aspirate was also noted. Feeding decisions, such as continuing, reducing, or withholding feeds, were based on the volume and characteristics of the aspirated gastric residuals, as per standard NICU protocols.

In the non-aspiration group, gastric residuals were not actively aspirated using a syringe. Instead, the orogastric or nasogastric tube was left open, allowing passive drainage of the gastric contents into a collection bag. This passive drainage was allowed to occur for a short period, typically a few minutes, before each feeding to prevent any build-up of gastric contents that could impede feeding. The volume and appearance of passively drained gastric residuals were not routinely measured or used to guide feeding decisions in this group. Instead, feeding continuation or modification was based on the clinical presentation of the infants and signs of feeding intolerance, such as abdominal distension or emesis.

Nurses in the non-aspiration group were provided with clear guidelines and training to ensure adherence to the study protocol. Regular audits and staff meetings were conducted to reinforce the importance of following the non-aspiration protocol and to address any concerns or questions raised by the nursing staff. In the event of any clinical concerns, such as signs of feeding intolerance or suspected NEC, nurses were advised to consult with the medical team and follow standard NICU protocols for managing these situations. If, in the clinical judgment of the medical team, aspiration of gastric residuals was deemed necessary for diagnostic or therapeutic purposes, this was documented as a protocol deviation, and the reasons for the deviation were recorded.

#### Data collection

Demographic data, medical history, feeding details, and clinical outcomes were extracted from medical records using a standardized data collection form. Gastric residual characteristics were recorded at each feeding. The diagnosis and staging of NEC were performed using Bell criteria, supplemented by laboratory tests, imaging, and clinical judgment. Data collection spanned only the initial hospitalization in the NICU. Long-term follow-up was not performed. All data were deidentified before analysis. The statisticians performing the data analysis were blinded to group allocation.

### Statistical analysis

All statistical analyses were performed with SPSS© version 26 (IBM Corp©, Armonk, NY, 2023). The level of significance was established at 0.05 for all analyses. Descriptive statistics, including frequencies, percentages, means, and standard deviations, were used to summarize the characteristics of the study population. Differences in baseline characteristics between the gastric residual aspiration and non-aspiration groups were compared using independent samples t-tests for continuous variables and the chi-square test for categorical variables. Differences in primary and secondary outcomes, such as incidence of NEC, feeding intolerance, time to full feed, etc., between the two groups were compared using independent samples t-tests for continuous variables and chi-square or Fisher’s exact test for categorical variables. Bivariate correlations between neonatal risk factors and NEC outcomes were examined using Pearson’s correlation coefficient. A multivariate binary logistic regression analysis was performed to determine risk factors independently associated with NEC. Variables with *p* < 0.25 in bivariate analyses were included in the multivariate model. Odd ratios and 95% confidence intervals were calculated. Model diagnostics were performed to check for multicollinearity and outliers. The general fit of the model was assessed using the Hosmer-Lemeshow goodness-of-fit test.

## Results

**Fig. 1:**
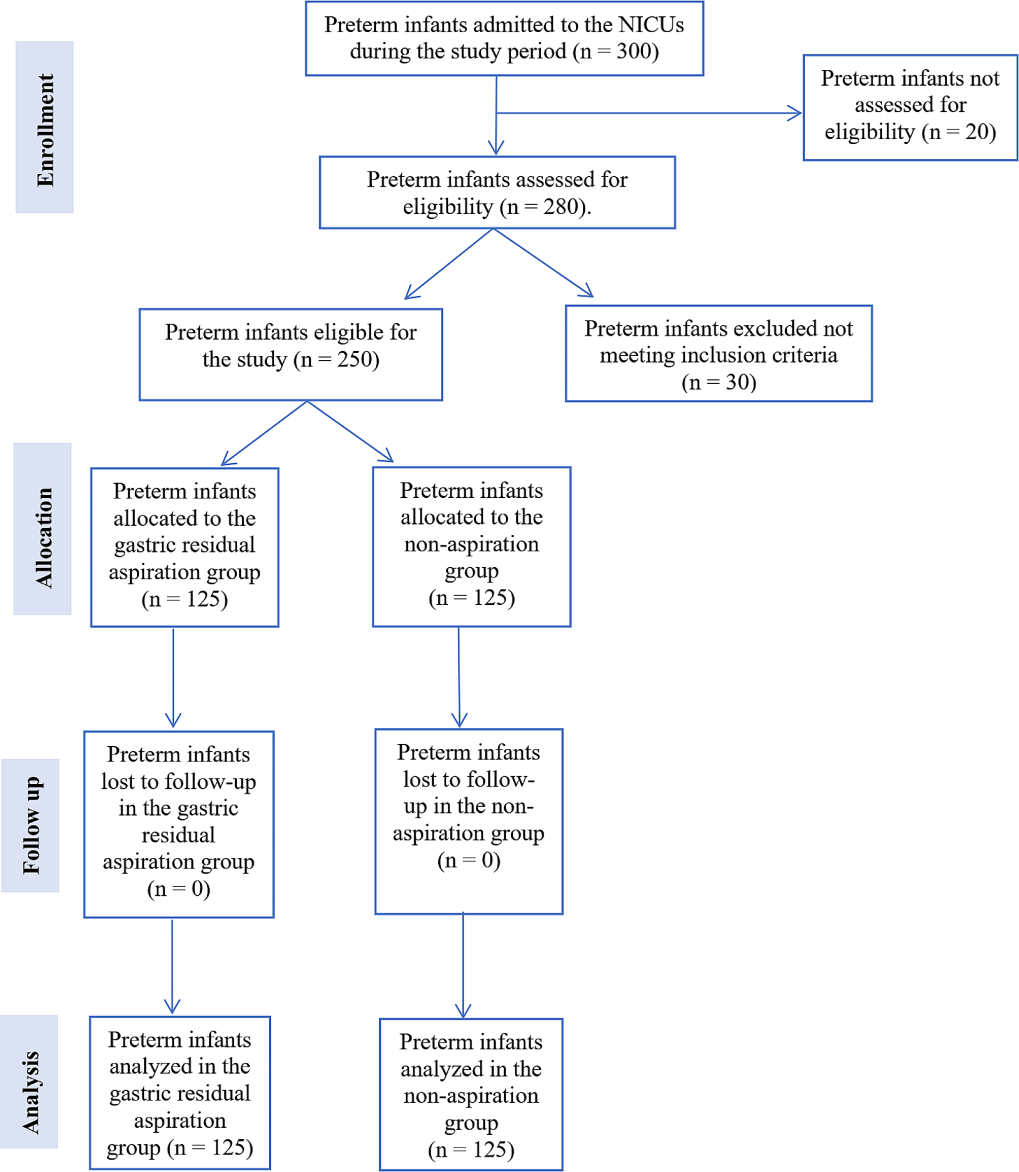
Study flow chart

Figure 1 presents the study flow chart, which summarizes the participant recruitment, allocation, follow-up, and analysis process. A total of 300 preterm infants were admitted to the NICUs during the study period. Of these, 20 infants were not assessed for eligibility due to not meeting the basic criteria for consideration (*n* = 12), being transferred or discharged before the eligibility assessment (*n* = 6), and parents declining participation before the eligibility assessment (*n* = 2). Of the 280 preterm infants assessed for eligibility, 30 were excluded. Among the excluded infants, 15 did not meet the inclusion criteria, and 15 met the exclusion criteria. The reasons for exclusion were intrauterine growth retardation (*n* = 5), respiratory distress (*n* = 4), circulatory instability (*n* = 3), suspected early-onset sepsis (*n* = 2), gastrointestinal tract malformations (*n* = 1), and other severe congenital malformations (*n* = 0). A total of 250 preterm infants were found eligible for the study and were allocated to either the gastric residual aspiration group (*n* = 125) or the non-aspiration group (*n* = 125). No infants were lost to follow-up in either group, resulting in a final sample size of 125 infants analyzed in each group.

Table 1 shows that the two study groups were well-matched at enrollment, with no significant differences in key baseline characteristics. The mean gestational age (28.5 ± 2.3 vs. 28.7 ± 2.1 weeks) and birth weight (1100 ± 150 vs. 1125 ± 140 g) were similar between the gastric residual aspiration and non-aspiration groups. The distribution of male sex (60% vs. 58%), maternal complications (30% vs. 28%), antenatal steroid use (55% vs. 53%), multiple births (25% vs. 24%), and location of the NICU (El Monira: 50% vs. 49%; El Manial: 50% vs. 51%) were also comparable between the groups. The incidence of common morbidities, including respiratory distress syndrome, patent ductus arteriosus, intraventricular haemorrhage, late-onset sepsis, and retinopathy of prematurity, was comparable between the gastric residual aspiration and non-aspiration groups. The similarity in baseline characteristics suggests that any differences in outcomes observed between the groups are less likely to be attributed to pre-existing differences in the study population, strengthening the internal validity of the study.

**Table 1:** Preterm infant characteristics of both groups at enrollment in percentage distribution (*n* = 250)

Characteristics	Gastric Residual Aspiration Group (*n* = 125)	Non-aspiration group (*n* = 125)
- Gestational age (weeks, mean ± SD):	28.5 ± 2.3	28.7 ± 2.1
- Birth weight (grams, mean ± SD):	1100 ± 150	1125 ± 140
- Male sex (%)	60%	58%
- Maternal complications (%):	30%	28%
- Antenatal steroids (%):	55%	53%
- Multiple births (%):	25%	24%
- Respiratory distress syndrome (%)	65%	63%
- Patent ductus arteriosus (%)	35%	33%
- Intraventricular hemorrhage (%)	20%	19%
- Late-onset sepsis (%)	15%	16%
- Retinopathy of prematurity (%)	10%	11%
- NICU location:		
El Monira (%)	50%	49%
El Manial (%)	50%	51%

Table 2 offers a focused view of feeding practices and results between the gastric residual aspiration and non-aspiration groups. Initial observations reveal that both groups follow comparable practices: a slight preference for oral feeding over nasal feeding and a moderate lean toward formula milk over breast milk. The average duration for the infants to reach full feed is almost similar between the groups, with only a minor difference of 0.2 days. Furthermore, the incidence of feeding problems is closely correlated between the two cohorts. Overall, this table underscores the consistency in feeding practices and outcomes across the two groups, further strengthening the study’s internal validity by ensuring that any outcomes can be attributed more confidently to the intervention (aspiration vs. non-aspiration) rather than differences in feeding practices.

**Table 2:** Feeding practices and outcomes of study participants (*n* = 250)

Characteristics	Gastric Residual Aspiration Group (*n* = 125)	Non-Aspiration Group (*n* = 125)
- Initial feeding method:		
Oral (%)	70%	68%
Nasal (%)	30%	32%
- Formula milk (%)	60%	58%
- Time to reach full feed (days, mean ± SD)	5.2 ± 1.1	5.4 ± 1.0
- Feeding problems encountered (% yes)	15%	14%

Table 3 compares the characteristics of gastric residuals between infants who underwent active gastric residual aspiration and those who did not. In the non-aspiration group, gastric residuals were not actively aspirated but were allowed to drain passively into a collection bag. The data presented for the non-aspiration group represent the volume and characteristics of these passively drained residuals. The mean volume of gastric residuals was slightly higher in the aspiration group (5 ± 5 ml) compared to the non-aspiration group (4 ± 4.5 ml), but the difference was not statistically significant (*p* = 0.12). Similarly, the percentage of infants with residuals stained with bile (40% vs. 38%) and the mean frequency of residuals (2.5 ± 1 vs. 2.4 ± 0.9) were comparable between the groups, without statistically significant differences (*p* = 0.15 and *p* = 0.10, respectively). These findings suggest that the practice of gastric residual aspiration did not significantly alter the volume, appearance, or frequency of gastric residuals in preterm infants.

**Table 3:** Comparison of gastric residual characteristics between infants undergoing gastric residual aspiration and those not undergoing aspiration (*n* = 250)

Characteristics	Gastric residual aspiration group	Non-aspiration group	*p*-value
- Volume of gastric residuals (ml, mean ± SD)	5 ± 5	4 ± 4.5	0.12
- Bile-stained residuals (%)	40%	38%	0.15
- Frequency of residuals (mean ± SD)	2.5 ± 1	2.4 ± 0.9	0.10

*In the non-aspiration group, gastric residuals were not actively aspirated using a syringe. The data presented for this group represent the volume and characteristics of the passively drained gastric residuals collected in a bag attached to the open orogastric or nasogastric tube

Table 4 presents the clinical outcomes and complications in preterm infants according to whether they underwent gastric residual aspiration or not. The duration of stay in the NICU was similar between the two groups, with a mean duration of 28 ± 5 days in the aspiration group and 27 ± 4.5 days in the non-aspiration group. The rates of hospital readmissions within 30 days (8% vs. 8.8%), mortality (4% vs. 4.8%), and other complications such as sepsis and respiratory issues (16% vs. 16.8%) were also comparable between the groups. These findings suggest that the practice of gastric residual aspiration did not significantly influence clinical outcomes or the incidence of complications in preterm infants.

**Table 4:** Clinical outcomes and complications in preterm infants based on gastric residual aspiration practice (*n* = 250)

Outcomes	Gastric Residual Aspiration Group (*n*, %)	Non-Aspiration Group (*n*, %)
- Length of stay in the NICU (days)	28 ± 5	27 ± 4.5
- Hospital readmissions (within 30 days)	10 (8%)	11 (8.8%)
- Mortality	5 (4%)	6 (4.8%)
- Other complications (e.g., sepsis, respiratory issues)	20 (16%)	21 (16.8%)

*Length of stay in NICU is defined as the duration of the initial hospital stay from birth to discharge, expressed in days. This does not include any subsequent home care or readmissions

Table 5 presents a multivariate regression analysis identifying potential risk factors for NEC in preterm infants. Gestational age and birth weight emerge as significant determinants, with earlier gestation and lower weight presenting an increased risk. Although not definitively significant, the practice of gastric residual aspiration suggests a possible association that deserves closer inspection. In contrast, other variables, such as maternal complications, feeding type, and location of the NICU, do not show a strong association with the development of NEC. The table underscores the intricate etiology of NEC and emphasizes the need to consider multiple factors that influence it holistically.

**Table 5:** Multivariate regression analysis on risk factors for NEC development

Variable	Odds ratio (95% CI)	*p*-value
- Gastric Residual Aspiration (yes vs. no)	1.20 (0.98–1.47)	0.08
- Gestational age (per week)	0.85 (0.78–0.93)	< 0.01
- Birth weight (per 100 g)	0.90 (0.85–0.96)	< 0.01
- Maternal complications (yes vs. no)	1.15 (0.95–1.40)	0.15
- Type of feeding (formula vs. breast milk)	1.10 (0.97–1.25)	0.12
- NICU location (El Monira vs. El Manial)	1.05 (0.53–2.09)	0.89

Table 6 compares the incidence and severity of necrotizing enterocolitis (NEC) between infants who underwent residual gastric aspiration and those who did not. Most of the infants in both groups did not develop NEC (85.6% in the aspiration group vs. 84.8% in the non-aspiration group). The incidence of suspicious NEC (Stage 1), definite NEC (stage 2) and advanced NEC (Stage 3) was similar between the groups, with no statistically significant differences, as evident from odds ratios close to 1 and p-values > 0.05. The overall risk of developing NEC was also comparable between the groups (14.4% vs. 15.2%, OR = 0.94, 95% CI: 0.48–1.84, *p* = 0.8). These findings suggest that the practice of gastric residual aspiration did not significantly influence the incidence or severity of NEC in preterm infants.

**Table 6:** Comparison of NEC incidence and severity between infants who underwent gastric residual aspiration and those who did not undergo aspiration (*n* = 250)

NEC Outcome	Gastric Residual Aspiration Group (*n* = 125)	Non-aspiration group (*n* = 125)	Odds ratio (95% CI)	*p*-value
- No NEC	107 (85.6%)	106 (84.8%)	-	-
- Suspicious NEC (Stage 1)	10 (8%)	9 (7.2%)	1.12 (0.44–2.82)	0.80
- Definite NEC (Stage 2)	6 (4.8%)	7 (5.6%)	0.85 (0.28–2.57)	0.77
- Advanced NEC (Stage 3)	2 (1.6%)	3 (2.4%)	0.66 (0.12–3.68)	0.63
- Overall NEC Risk	18 (14.4%)	19 (15.2%)	0.94 (0.48–1.84)	0.8

Table 7 explores the association between feeding practices and the development of necrotizing enterocolitis (NEC) in preterm infants who underwent residual gastric aspiration compared to those who did not. The percentage of infants with oral initial feeding (60% vs. 58%), time to full feed > 5 days (35% vs. 33%), use of formula milk use (60% vs. 58%), and feeding problems (30% vs. 28%) were similar between the two groups. The odds ratios for these feeding parameters were close to 1, and the p-values were > 0.05, indicating that there were no statistically significant differences in the association between feeding practices and the development of NEC based on the practice of gastric residual aspiration. These findings suggest that the feeding practices examined in this study did not significantly influence the relationship between gastric residual aspiration and NEC in preterm infants.

**Table 7:** Comparison of feeding practices and their association with NEC between gastric residual aspiration and non-aspiration groups (*n* = 250)

Feeding Parameter	Gastric Residual Aspiration Group (*n* = 125)	Non-aspiration group (*n* = 125)	Odds ratio (95% CI)	*p*-value
- Oral initial feeding (%)	60%	58%	1.08 (0.93–1.26)	0.29
- Time to full feed > 5 days (%)	35%	33%	1.10 (0.96–1.27)	0.18
- Formula milk (%)	60%	58%	1.09 (0.94–1.28)	0.24
- Feeding problems (%)	30%	28%	1.11 (0.97–1.29)	0.13

## Discussion

This quasi-experimental study offered valuable information on the relationship between routine gastric residual aspiration and necrotizing enterocolitis (NEC) development in preterm infants. The findings suggest that aspiration of gastric residuals before feeding may not directly alter the risk or severity of NEC.

### Feeding practices and residual characteristics

A thorough analysis of the core of the study revealed fascinating insights into food practices and residual gastric properties. The mere act of gastric residual aspiration does not appear to profoundly affect feeding behavior, residual volume, or consistency. Our findings are in contrast to those of [69], who found that routine gastric residual aspiration was associated with a delay in the time to reach full enteral feeding in preterm infants. In their randomized controlled trial, infants in the aspiration group took longer to achieve full feeds compared to those in the no-aspiration group (median 11 days vs. 9 days, *p* = 0.01). The authors suggested that the practice of routine gastric residual aspiration may disrupt the normal gastrointestinal tract development and lead to feeding intolerance.

Residual gastric aspiration could affect the progression of feeding in preterm infants. On the other hand, our findings are consistent with those of [50], who found no significant differences in feeding practices between infants undergoing gastric residual aspiration and those who did not. Although it is tempting to view gastric residuals as mere indicators of food intolerance, one might wonder if their role extends beyond this. They may be early signs or precursors to more severe conditions such as NEC.

### No difference in NEC incidence with aspiration

Our central finding was that the incidence of NEC did not differ significantly between the gastric aspiration and non-aspiration groups. Approximately 15% of the infants developed some stage of NEC in both arms. Previous studies have been inconsistent, with some noting a higher NEC with aspiration [61, 65] while others did not find any difference [50–53]. Although the multivariate analysis (Table 5) suggested a possible association between gastric residual aspiration and NEC, with an odds ratio of 1.20 (95% CI: 0.98–1.47), the relationship did not reach statistical significance at the conventional 0.05 level (*p* = 0.08). It is important to note that the confidence interval for the odds ratio was wide and included 1, indicating that the true effect size could be smaller or larger than the point estimate. Furthermore, the interpretation of our results should be made with caution, given the limitations of our study design and sample size. Larger, well-controlled studies are needed to more definitively establish the relationship between gastric residual aspiration and NEC in preterm infants. While our findings suggest a possible association, they do not provide conclusive evidence that routine aspiration intrinsically increases the risk of NEC.

However, the etiology of NEC is complex. Although aspiration did not emerge as an independent risk factor in our regression analysis, elements such as lower gestational age and birth weight did. This agrees with [70], confirming the multifactorial nature of NEC. Our findings add to the evidence that routine gastric aspiration does not appear to directly precipitate the onset of NEC in preterm infants, but many factors are at play.

### No difference in NEC severity with aspiration

Furthermore, we analyzed the outcomes of NEC by stage severity. The percentages of infants with mild, moderate, or advanced NEC were comparable. Previous studies have not examined the severity in detail [51, 69]. This suggests that routine aspiration practice may not worsen progression or exacerbate NEC once present. However, larger samples are needed to confirm this due to the low frequency of advanced NEC.

The lack of difference in NEC severity between the aspiration and non-aspiration groups suggests that routine gastric residual aspiration may not be an effective strategy for mitigating the severity of NEC once it develops. This finding may have implications for the management of preterm infants with suspected or confirmed NEC.

### No major impact of clinical outcomes on aspiration

Longitudinal stay, readmission, mortality, and other complications were similar regardless of gastric aspiration. Some researchers have proposed that aspiration can delay feeding progress and prolong hospitalization [71]. However, we found minimal differences in clinical outcomes. However, no long-term follow-up was conducted; potential outcomes remain unknown.

The mean length of stay in the NICU of approximately 27–28 days for infants with a mean gestational age of 28.5 weeks in our study is shorter than what has been reported in some other studies. This difference may be attributed to variations in clinical practices, discharge criteria, and the availability of local resources and support services. It is important to note that the length of stay reported in our study represents only the initial hospital stay and does not include any subsequent home care or readmissions. Future studies should consider exploring factors that influence the duration of hospital stay for preterm infants in different settings and the potential impact of these variations on long-term outcomes. While shorter hospital stays may be beneficial for infants and their families, it is crucial to ensure that early discharge does not compromise the provision of appropriate care and support for preterm infants at risk of developing NEC or other complications.

### No difference in residual volume/frequency with aspiration

Interestingly, residual volume and frequency did not differ significantly between the aspiration and non-aspiration groups. Some have hypothesized that aspiration could alter gastric motility and residuals [72], but we did not find significant effects. A minimal comparison of residuals between groups has been studied. While aspiration did not appear to impact residual characteristics, the clinical utility of measuring volumes warrants further scrutiny.

### Limitations, future directions, and contrasting perspectives

Our study has several limitations that should be considered when interpreting the results. The quasi-experimental design, which lacks randomization, may have introduced selection bias and limited the generalizability of our findings. Furthermore, our study may have been subject to other biases, such as patient variability and nurses’ expertise in performing routine gastric aspiration. To address these limitations, future studies should employ randomized patient selection, standardized protocols for gastric residual aspiration, and stratification of patients based on key characteristics.

Although our study sheds light on the potential non-significance of gastric residual aspiration in the context of NEC, it is paramount to approach this insight with caution. The multifaceted nature of NEC underscores the likelihood that a confluence of factors, rather than a singular determinant, dictates its onset and progression. Moreover, while our findings are comprehensive, they do not provide a conclusive argument for the clinical relevance of gastric residual aspiration. Some might argue that, while aspiration may not directly influence NEC, its indirect implications, such as affecting the gut microbiota, cannot be overlooked. Future studies should also explore the potential indirect effects of gastric residual aspiration on the development of NEC, such as its impact on the gut microbiome and immune function, as these factors may play a crucial role in the pathogenesis of NEC.

The importance of evidence-based feeding protocols in reducing the incidence of NEC in preterm infants has been highlighted in a recent systematic review by Jasani and Patole [73]. The authors found that standardized feeding regimens, which include strategies such as slow advancement of enteral feeds and the use of human milk, can significantly reduce the risk of NEC in this vulnerable population. Our findings and the evidence presented in this systematic review underscore the need for neonatal healthcare providers to adopt and adhere to evidence-based feeding protocols to improve outcomes for preterm infants.

In conclusion, while our study suggests that routine gastric aspiration may not alter NEC or clinical outcomes in preterm infants, we caution against generalized conclusions given the multifaceted nature of NEC etiology. More data are needed to shape evidence-based feeding guidelines and improve neonatal care.

## Conclusions

This quasi-experimental study provides valuable information on the relationship between gastric residual aspiration and necrotizing enterocolitis (NEC) among preterm infants. Our findings suggest that aspirating gastric residuals before feeding may not substantially alter the risk or severity of developing NEC. Several crucial observations support this conclusion. First, the gastric residual aspiration and non-aspiration groups in this study exhibited remarkably similar baseline characteristics. This homogeneity in gestational age, birth weight, feeding practices, and other variables lends credibility by minimizing potential confounders. Furthermore, the mere act of aspirating gastric residuals did not appear to profoundly affect residual volume, consistency, feeding progression, or other results. More importantly, the overall incidence and severity of the staging of NEC were comparable between the two groups.

Our regression analysis revealed that lower gestational age and birth weight played a more pronounced role in NEC development than gastric aspiration. This is consistent with other recent research demonstrating the complex and multifactorial nature of the etiology of NEC. While our study provides evidence to downplay the importance of routine gastric aspiration, it is only one piece of the complex puzzle behind the pathogenesis of NEC. Based solely on these findings, we cannot conclusively determine that gastric residuals lack clinical utility. However, this research highlights the need to holistically examine the multiple risk factors contributing to NEC using robust multicenter studies. In summary, although gastric residuals remain important clinical markers, our findings call into question the premise that routine aspiration directly reduces the risk of NEC in preterm infants. This has implications for the development of evidence-based nutrition guidelines. More research is needed incorporating advances in neonatal care to deepen our understanding of the etiology and prevention of NEC.

## Data Availability

Data will be available upon the corresponding author’s request.

## Abbreviations

NEC: Necrotizing Enterocolitis
NICU: Neonatal Intensive Care Unit
IRB: Institutional Review Board
GRV: Gastric Residual Volume
SD: Standard Deviation
CI: Confidence Interva
SPSS: Statistical Package for the Social Sciences
